# Unveiling the hidden viromes across the animal tree of life: insights from a taxonomic classification pipeline applied to invertebrates of 31 metazoan phyla

**DOI:** 10.1128/msystems.00124-24

**Published:** 2024-04-23

**Authors:** Pau Alfonso, Anamarija Butković, Rosa Fernández, Ana Riesgo, Santiago F. Elena

**Affiliations:** 1 Instituto de Biología Integrativa de Sistemas (CSIC-Universitat de València), Paterna, València, Spain; 2 Institut Pasteur, Université Paris Cité, CNRS UMR6047 Archaeal Virology Unit, Paris, France; 3 Instituto de Biología Evolutiva (CSIC-Universitat Pompeu Fabra), Barcelona, Spain; 4 Museo Nacional de Ciencias Naturales (CSIC), Madrid, Spain; 5 Department of Life Sciences, Natural History Museum of London, London, United Kingdom; 6 The Santa Fe Institute, Santa Fe, New Mexico, USA; Dalhousie University, Halifax, Nova Scotia, Canada

**Keywords:** viromes, invertebrates, metatranscriptomics, virus ecology, compositional analysis

## Abstract

Invertebrates constitute the majority of animal species on Earth, including most disease-causing agents or vectors, with more diverse viromes when compared to vertebrates. Recent advancements in high-throughput sequencing have significantly expanded our understanding of invertebrate viruses, yet this knowledge remains biased toward a few well-studied animal lineages. In this study, we analyze invertebrate DNA and RNA viromes for 31 phyla using 417 publicly available RNA-Seq data sets from diverse environments in the marine-terrestrial and marine-freshwater gradients. This study aims to (i) estimate virome compositions at the family level for the first time across the animal tree of life, including the first exploration of the virome in several phyla, (ii) quantify the diversity of invertebrate viromes and characterize the structure of invertebrate-virus infection networks, and (iii) investigate host phylum and habitat influence on virome differences. Results showed that a set of few viral families of eukaryotes, comprising *Retroviridae*, *Flaviviridae*, and several families of giant DNA viruses, were ubiquitous and highly abundant. Nevertheless, some differences emerged between phyla, revealing for instance a less diverse virome in Ctenophora compared to the other animal phyla. Compositional analysis of the viromes showed that the host phylum explained over five times more variance in composition than its habitat. Moreover, significant similarities were observed between the viromes of some phylogenetically related phyla, which could highlight the influence of co-evolution in shaping invertebrate viromes.

**IMPORTANCE:**

This study significantly enhances our understanding of the global animal virome by characterizing the viromes of previously unexamined invertebrate lineages from a large number of animal phyla. It showcases the great diversity of viromes within each phylum and investigates the role of habitat shaping animal viral communities. Furthermore, our research identifies dominant virus families in invertebrates and distinguishes phyla with analogous viromes. This study sets the road toward a deeper understanding of the virome across the animal tree of life.

## INTRODUCTION

Viruses are arguably the most successful biological entities on Earth, given their ubiquitousness, diversity, and numerical abundance, apart from their capability to infect organisms from all domains of cellular life (1–3). In recent times, high-throughput sequencing technologies have catalyzed the identification of new viruses, and numerous studies have leveraged metagenomics and metatranscriptomics to perform discovery of novel viruses at a massive scale (4–10). Viruses identified through these approaches enrich reference databases and even are incorporated into the taxonomy of the International Committee on Taxonomy of Viruses (ICTV) (11, 12), contributing to a better comprehension and representation of the virosphere.

Thanks to these methods, our understanding of invertebrate viromes, which constitute a vastly more diverse and abundant group when compared to vertebrates, is ongoing unprecedented expansion (13). However, despite the accelerated pace of virus discovery through metagenomics and metatranscriptomics, knowledge of animal viral diversity is still skewed toward viruses that cause disease in humans, involve arthropod vectors, or affect economically relevant species, including culturable arthropods and mollusks (14). Countless viral species, which might play pivotal roles in underexplored organisms, still remain obscured by this bias (15).

The scientific community is actively making efforts to address this bias. In addition to more conventional studies on disease-vector arthropods (16–20), researchers have begun to characterize the viromes of sponges (21, 22), cnidarians (23–25), soil-inhabiting nematodes (26), and echinoderms (27). Some works have greatly improved the exploration of invertebrate viromes with a larger scope, simultaneously analyzing the viromes of invertebrate species distributed across different phyla, including the first characterizations of annelid, platyhelminth, and tunicate viromes (4, 28). However, the metazoa tree of life encompasses a vast diversity of groups (29), and viral communities have yet to be discovered for the first time in many phyla (14).

The Sequence Read Archive (SRA; https://www.ncbi.nlm.nih.gov/sra) hosts millions of publicly accessible metatranscriptomic and metagenomic data sets that comprise more than 7⋅10^16^ sequenced bases (https://www.ncbi.nlm.nih.gov/sra/docs/sragrowth/). Because many researchers who create these data sets do not focus on viral communities, these data provide an opportunity to explore virus diversity in uncharted organisms and ecosystems (30). The insightfulness of such analyses depends on the quality of complementary metadata in the databases (31), including accurate descriptions of sampling conditions, geographic location, and host taxonomy, among other factors. In the present study, the viromes of 417 publicly accessible invertebrate RNA-Seq data sets spanning 31 animal phyla are inferred through a read taxonomic classification pipeline. After classifying the read sequences to existing families of RNA and DNA viruses, the study (i) provides the first description of the virome composition at the family level for several invertebrate phyla, such as Bryozoa, Ctenophora, or Onychophora; (ii) involves diversity measurements for the estimated viromes and the characterization of the obtained virus-invertebrate interaction network structure; and (iii) investigates whether virome composition differences among samples can be attributed to the host phylum and/or habitat.

## MATERIALS AND METHODS

### Sampling of sequencing data sets

All sequencing accession numbers for the 31 different invertebrate phyla stored in NIH’s SRA database were retrieved in January 2023. This comprehensive database combines information from NCBI’s SRA, EBI’s European Nucleotide Archive (ENA), and DNA Data Bank of Japan (DDBJ) databases, according to the International Nucleotide Sequence Database Collaboration (INSDC) initiative. This added up to a total of 762,540 accessions for which metadata from multiple fields were fetched from SRA and BioSample databases. Mainly, it was important to collect for each accession its associated BioSample identifier, the organism’s TaxID, the number of sequenced bases, the average read length, and details about the library, such as its strategy, source, and layout. Additionally, characteristics of the sampling conditions were retrieved from isolation_source, geo_loc_name, and lat_lon fields from BioSample data.

The taxonomic lineage of each TaxID, spanning from the phylum level to subspecies, was obtained using TaxonKit and the NCBI’s taxonomy database (taxdump) version last modified on 2 March 2023. Because NCBI’s species names often involve subspecies, laboratory strains, or species mixtures, it was necessary to perform a harmonization step. Namely, all species names had to be binomial, and when this degree of specificity could not be reached, the “sp.” or “spp.” abbreviations would be added after the lowest certain taxon.

SRA metadata information was used to narrow down the selected accession data to a subset that was eligible for the analysis. The following three criteria were used to do this: (i) library strategy was restricted to RNA-Seq, (ii) average read length had to be greater or equal than 75 bases, and (iii) the total number of sequenced bases had to be greater than 3 Gbases. The rationale behind these last two criteria is to obtain samples with longer reads to have higher sensitivity of read-based taxonomic classifiers and to keep samples where the virome composition estimation is as independent from the sequencing depth as possible, respectively. This led to a total number of 82,145 eligible run accessions. Moreover, by using the three abovementioned criteria, a decline was noted in the number of unique species to roughly one-fourth of the original number.

Only one run per species was included for the analysis, because the occurrence of multiple runs per species could introduce potential bias. In that case, a phylum could be represented by few species with multiple samples, narrowing the phylogenetic diversity of hosts that is explored within that phylum and potentially underestimating the diversity of its viromes. Furthermore, because the distribution of unique species is highly asymmetrical between phyla, the maximum number of analyzed species was set to 20 per phylum. Each species had to include at least one accession that passed the selection criteria to be sampled. However, some phyla were left with a smaller number of samples due to a lack of species with eligible accessions. In summary, the sampling algorithm tries to maximize the number of samples selected for rare phyla (i.e., phyla with a smaller number of data sets), while maintaining a balanced number of samples for those phyla with a greater availability of data sets. However, an exception was made for mollusks (39 observations), because they contained a large number of unique species from a wide range of habitats. Taking this into account, the selected species, as well as their final run accession for the analysis, were sampled randomly. Samples that were sequenced with less than 30 Gbases were prioritized over larger data sets in order to speed up the analysis. Namely, data sets with more than 30 Gbases were only included when 20 samples per phylum could not be reached otherwise.

The habitat of each organism was reconstructed using the descriptors about the location and source of each sample’s isolate alongside the information found in the corresponding WoRMS website (32) entry. Habitat annotation for 143 samples relied solely on ecological information of the species obtained from WoRMS or specific publications, because the geographical location was unrecoverable from their metadata in SRA and BioSample. We classified each sample’s habitat into five categories: brackish, freshwater, intertidal zone, marine, and terrestrial. This also served as a way to identify samples that were laboratory-reared, finding that nine out of the 417 analyzed accessions had a laboratory origin. This fraction could be even larger due to the widespread sparse metadata annotation.

### Downloading and pre-processing of raw read files

Raw read files were downloaded in FASTQ format using fasterq-dump of SRA toolkit version 2.11.2 (https://github.com/ncbi/sra-tools). A sequential three-step pre-processing phase was conducted over the downloaded raw reads.

First, clumpify.sh script of BBMap package version 38.96 (https://sourceforge.net/projects/bbmap/) was run to remove exact duplicate reads. These can be caused by technical issues of NGS and do not reflect the true abundance of the transcript within the sample’s RNA molecule population. This step was implemented with the purpose of reducing the volume of reads for downstream analyses, which speeds up the pipeline.

Second, SortMeRNA version 4.3.4 (33) was used to filter out reads of abundant transcripts with non-viral origin. All reads were compared against SortMeRNA’s default rRNA databases for 18S and 28S rRNA subunits of eukaryotes and a custom database consisting of more than 4,000 variants of cytochrome c oxidase (*COX*) mRNA sequences. If a single-end read showed significant similarity to any sequence in these reference databases, it was excluded. Similarly, paired-end reads were only kept for downstream analyses if neither of the ends had a match with any sequence in the reference databases.

Third, Trimmomatic version 0.39 (34) was used over the remaining reads to trim out adapters introduced by NGS platforms and terminal regions with an average base call accuracy lower than 80%. The minimum read length after trimming was set to 25.

In some instances, pairing of FASTQ files was found to be corrupted either from the beginning or after any of the pre-processing steps. When necessary, pairing was restored using BBMap’s repair.sh script to resume the pipeline.

### Read-based taxonomic classification

The taxonomic classification of pre-processed reads was conducted using Kaiju version 1.8.2 (35). A subset of NCBI’s RefSeq NR proteins database, which includes all protein sequences classified under the superkingdom “Viruses” (TaxID = 10239), served as the reference database. This subset was derived from a version of the NR proteins database downloaded in November 2022.

Kaiju takes the reads and translates them to all six possible reading frames. Subsequently, these amino acid sequences are compared with those in the reference database. The best match was stored only if the alignment reached an *E* value < 10^−5^ threshold.

When a read has a significant viral match, it is added to the count of its particular viral family. However, if the virus of the matching sequence is not classified to a taxon as specific as the family level or if it is not classified under an existing viral family, the read count is added to a category of viruses with uncertain family. So, a vector of read absolute abundances of viral families is constructed for each sample and these are combined into a matrix.

It is important to acknowledge that the virus family nomenclature used in this study is the one reported on the NCBI’s taxonomy database. This database does not incorporate recent updates that have been determined by the ICTV. As a result, it may lack the most recently approved names or, alternatively, include family names that have already been discouraged by the ICTV.

### Statistical analysis of ecological diversity metrics

α-diversity at the family level, which refers to the richness of viral families, and Shannon’s diversity index (*H*′) were computed for all estimated viromes restricted to eukaryotic families. α-diversity was calculated as the number of viral families with positive read counts for each sample. *H′* was calculated for each sample *i* as $H_{i}`=-\sum_{j=1}^{F_{i}}p_{ij}logp_{ij}$, where *F_i_* is the number of families found in sample *i*, and *p_ij_* is the proportion of viral reads that are assigned to family *j* in sample *i*.

To find differences between phylum and habitat groups, analysis of variance (ANOVA) models were fit separately for both of these univariate diversity metrics. To ensure sufficient group sizes, the data set was reduced by excluding phyla and habitats represented by less than five observations. For both α-diversity and *H*′, the best model was selected with the criterion of minimizing the Akaike information criterion (AIC) after considering all possible combinations of phylum and habitat as covariates.

As group sizes were unbalanced for both factors, models were checked for homoscedasticity with Levene’s test. In the case of *H*′, phylum grouping was seen to introduce heteroscedasticity in the model, so the data were transformed using the inverse of the Box-Cox transformation of *H*′ with parameter λ = 2.5 before the model was fit.

Tukey’s post hoc tests were conducted over the selected models using the phylum as covariate to identify which phyla were responsible for driving the observed differences, if any.

Several additional metrics from the field of community ecology were computed after transforming the counts matrix to relative abundances, which indicate the intensity of the ecological interactions. Also, the bipartite infection network was restricted to the 30 eukaryotic viral families that reached an average relative abundance higher than 0.01% among the samples.

At the network level, measures of (i) linkage density, (ii) *H*_2_*′* specialization index (36), (iii) nestedness using the method described by Almeida-Neto et al. (37), and (iv) *Q* modularity index (38) were calculated. One thousand null models were run to simulate estimates of these four indices under randomness of interactions. The sampling distributions of the simulated indices were used to perform *z*-tests for the observed values. In the case of indices with closed intervals of possible values (ii–iv), it was checked that the observed value was separated enough from the boundaries to avoid the violation of the normality assumption.

At the level of individual viromes, the *d*′ specialization index (36) was calculated and used to fit an ANOVA model grouping *d*′ estimates by the host’s phylum analogous to the ones described above. *d*′ was also calculated for each viral family in the matrix.

### Virome composition analysis

The absolute abundances matrix, which was originally measured in read counts, was first restricted to families of eukaryotic viruses. The vector of abundances of each sample can be regarded as a composition, where each viral family constitutes a part of the virome. In recent years, it has been suggested that microbiome, and by extension virome, data sets should be analyzed under the framework of compositional data analysis (39).

These methods require a processing of the abundance matrix previous to the analysis. In this case, compositions were first transformed to relative abundances vectors with respect to the total number of reads assigned to eukaryotic viral families. In the case of microbiome compositional data, it has also been acknowledged that dimensionality reduction both in observations and features can lead to a more informative analysis (40). In this study, it was decided to exclude viral families that did not reach an average relative abundance of 0.01%. Then, imputation of zero values was conducted to avoid the occurrence of null values in the compositions. Finally, the resulting imputed vectors of relative abundances were transformed to centered log-ratio (*clr*) coefficients.

After applying the transformation, the data set was reduced to the subset of phyla with 15 or more samples. The matrix of Euclidean distances for this subset of *clr*-transformed compositions was used to fit permutational analysis of variance(PERMANOVA) models (41). Additionally, permutational multivariate analysis of dispersion (PERMDISP) (42) tests were performed alongside PERMANOVA to further investigate the occurrence of centroid location and within-group dispersion differences in the virome compositions between groups. Different PERMANOVA models combining phylum and habitat as covariates were fit and compared in terms of AIC to select the best model.

### PERMANOVA bootstrap analysis of differences between virome compositions

Using the model formula selected after the process detailed in the previous section, a bootstrap implementation of PERMANOVA is proposed to test the existence of differences between the virome composition centroid locations for all pairs of phyla in the data set.

The use of unbalanced group sizes is known to cause issues when fitting PERMANOVA models (43). However, group sizes are heterogeneous in this study. In an effort to use all available data and keep balanced group sizes, 1,500 bootstrap iterations were run using a resampling algorithm with the following steps: (i) sample 15 observations per phylum allowing for replacement, (ii) impute a new phylum for a random observation (if there are duplicate observations, replace the true phylum in all of them), (iii) exclude observations from the imputed phylum until a group size of 15 is reached, making sure the shuffled observation(s) is kept, (iv) sample new observations for the shuffled observation’s old phylum to restore the balanced group sizes for all phyla (this step is restricted to not sample again the shuffled observation), (v) transform the absolute abundance matrix to a matrix of *clr* coefficients, (vi) PERMANOVA models and PERMDISP tests are performed for every pairwise combination of phyla, ignoring self and symmetrical comparisons. This results in *n*(*n* – 1)/2 combinations per bootstrap iteration, where *n* is the number of phyla. For each combination, the resulting *P* values of PERMANOVA and PERMDISP are computed and adjusted by the false discovery rate (FDR) method considering the total number of tests per iteration. These results are discretized based on the FDR being greater or lower than 0.05. Finally, (vii) the combination of acceptance/rejection of the hypotheses of both tests are encoded by a factor of four levels that is stored for each iteration and combination of phyla in an array before proceeding to the next iteration.

### Statistical analysis software

R version 4.0.4 was used for all the statistical analyses downstream of the bioinformatic pipeline. R programming was carried out using RStudio version 2022.12.0.

In the analysis of species-level ecological measures, the implementation of Levene’s test in car package (version 3.1.2) (44) was used to check the assumption of homoscedasticity of ANOVA models. Library multcompView (version 0.1-9) (45) was used to obtain the compact letter display groups for the results of Tukey tests. Also, library MASS (version 7.3-60) (46) was used to perform the Box-Cox transformation needed for the *H*′ ANOVA model.

The calculation of other measures of community ecology both at network level and at invertebrate virome level was performed entirely using the R library bipartite (version 2.18) (47). Null models were simulated using the “r2d” method for quantitative matrices implemented in nulls() function. Both observed and simulated values of the indices were calculated using (i) networklevel() with method “linkage density” for the linkage density estimate, (ii) H2fun() for the overall specialization index, (iii) nested() with method “NODF2” for the nestedness, and (iv) computeModules() for the *Q* modularity index. *d′* specialization index was computed using dfun().

In the compositional analysis, libraries zCompositions (version 1.4.0) (48) and compositions (version 2.0.4) (49) were used to prepare the *clr* coefficients matrix with functions cmultRepl() using method “CZM” for the replacement of null values and clr() to perform the *clr* transformation, respectively. Functions adonis() and betadisper() of R library vegan (version 2.6-4) (50) were used to fit PERMANOVA models and perform PERMDISP tests, respectively.

Graphics were generated using R base, ggplot2 (version 3.3.5) (51), bipartite, heatmap3 (version 1.1.9) (52), and igraph (version 1.2.8) (53) packages.

## RESULTS AND DISCUSSION

### Characteristics of the RNA-Seq data sets

In the obtained collection of RNA-Seq data sets, the average sample had 8.8 Gbases sequenced. Twenty-two percent of the samples exceeded 10 Gbases, while the rest of the samples were distributed within the range of 3 to 10 Gbases. With respect to read lengths, approximately 12% of the samples had an average read length spanning 75 to 100 bases, and 27% featured longer average read lengths exceeding 150 bases. Thus, the majority of samples were within the range of 100 to 150 bases per read. Furthermore, the transcriptomes were obtained using single-end libraries only for 15 samples, while the rest were based on paired-end sequencing.

Data sets were generated with Illumina Genome Analyzer (14), HiSeq (314), MiSeq (11), NextSeq (36), and NovaSeq (41) equipment, with the exception of a single sample generated with PacBio Sequel II (SRR18516523). This sample was kept for all parts of the analysis, because its estimated virome was not seen to be outlying. As for the library selection methods, viral particle enrichment was not declared for any of the samples, because in most cases, the goal of the data sets was phylogenomics or the characterization of the animal’s transcriptome. Selection of molecules with polyA was the most common selection method (255 samples), followed by random priming (132 samples). In the case of libraries with polyA selection, non-polyadenilated RNA molecules of viruses could have been filtered out.

**Fig 1:**
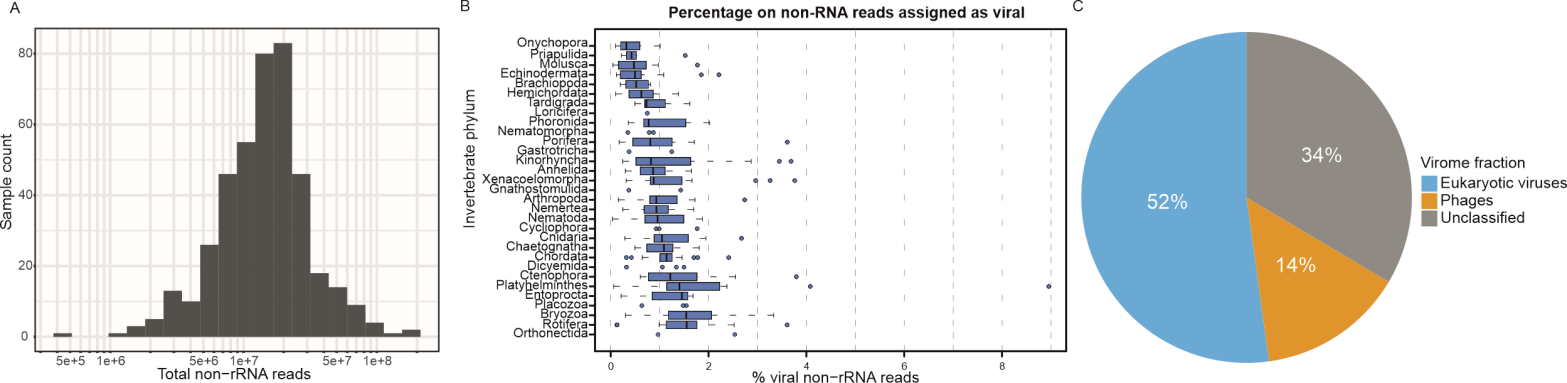
Overview of the bioinformatics pipeline results. (A) Logarithmic scale plot for the distribution of the total number of reads remaining after pre-processing for each sample. These are the sets of reads that are used as input for the taxonomic classification step. (B) Distribution of the percentage of reads that showed significant similarity to any viral protein sequence in the *nr* database. (C) Average percentages of viruses by host domain in the whole data set. Host inferences were made based on the viral family annotation of each hit. The unclassified fraction of the virome comprises viruses of uncertain family.

Pre-processing produced a decline in the total number of reads of at least 45% in all samples. As a result, the number of reads used as the source for taxonomic classification ranged between 5 and 50 million for 86% of the samples (Fig. 1A). More detailed information about the pre-processing results is available in the supplemental materials at https://git.csic.es/sfelenalab/invertebrates-virome/-/tree/main/Supplementary%20files.

### Taxonomic distribution of the selected samples

**Fig 2:**
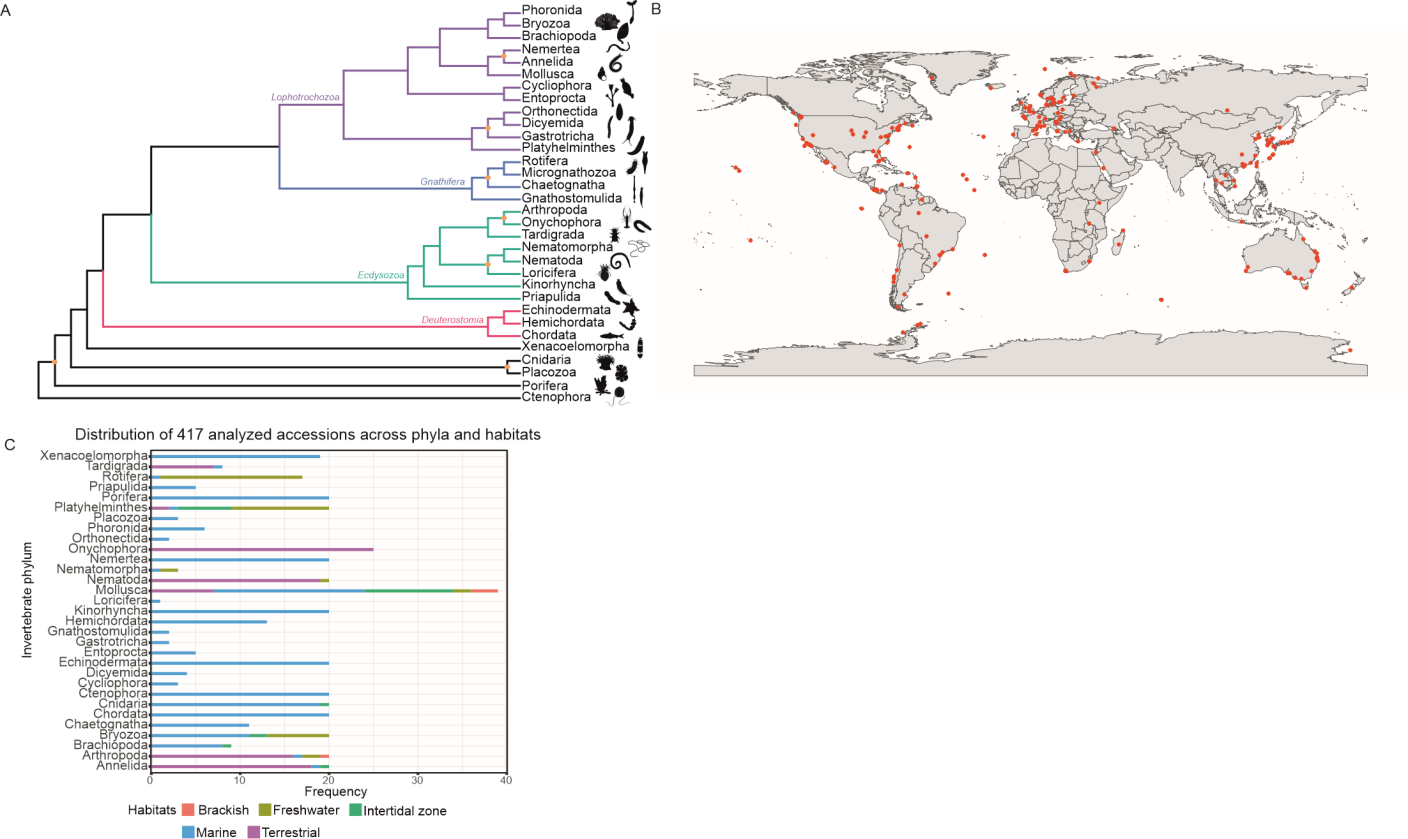
Diversity overview of the invertebrate samples. (A) Approximate topology of the animal tree of life based on our current understanding on animal phylogenetic interrelationships. Polytomies were arbitrarily avoided, opting instead for presentation of nodes with low support or conflicting information across the literature with orange circles. (B) Geographical locations of the analyzed samples. Latitude and longitude coordinates were originally available for 105 accessions and were approximately reconstructed for further 169 accessions using information from other metadata fields. (C) Distribution of selected samples across different animal phyla and habitats. Sampling depth was limited to 20 species for all phyla, except for Mollusca and Onychophora.

The sampling depth of the different invertebrate phyla exhibited significant variability (Fig. 2C). Nine phyla were represented by five or fewer samples, while 16 out of 31 phyla were each represented by at least 15 samples of unique invertebrate species. This variation is due to the differences in the number of species with publicly available transcriptomic data sets (see Table S1 at https://git.csic.es/sfelenalab/invertebrates-virome/-/tree/main/Supplementary%20files). Two main factors contribute to this: (i) the attention and research focus given to certain species based on anthropocentric interests and (ii) the existing biodiversity within each phylum. In fact, it is noteworthy that 83% of all described invertebrate species as of 2013 belonged to the phylum Arthropoda (54). In the same report, it was stated that species of Annelida, Arthropoda, Bryozoa, Cnidaria, Echinodermata, Mollusca, Nematoda, Platyhelminthes, and Porifera collectively accounted for 98.6% of all invertebrate species.

A phylogenetic tree for the animal tree of life is presented in Figure 2A to assist in the interpretability of the results. The topology of this tree is mainly based on the results of Laumer et al. (55). However, in order to include the full set of 31 phyla considered for this study, other works that specifically addressed the placement of certain phyla were taken into account, which was the case for Kinorhyncha, Loricifera, and Nematomorpha (56), and for Dicyemida and Orthonectida (57). Certain nodes that are proposed only in new studies, supported by relatively low bootstrap values, or that are in contradiction between different analyses in the literature were highlighted as still disputed/problematic (58–63), as this tree is presented for guidance only.

### Habitat distribution of selected samples

Geographically, the chosen samples covered a wide range of locations around the world, with a notable preference for Europe, North America, Eastern Asia, and Australia (Fig. 2B), although it should be noted that the observed spatial distribution across the globe could have been biased by the lack of coordinates for roughly a third of the samples. Besides their ecological habitat, geographic location is acknowledged to shape virome compositions of invertebrates. For instance, a recent study comparing viromes of marine annelids, arthropods, and mollusks across different seas found sea-specific virus community profiles (64). Therefore, it is crucial to avoid the over-representation of specific locations before drawing conclusions at a global scale.

With regard to the habitat imputations, sampling depth was also largely variable by habitat (Fig. 2C). The marine habitat constituted the most abundant category with 257 samples, followed by the terrestrial habitat with 94 observations. The freshwater habitat was less common, with 41 samples, while the intertidal zone and brackish habitats were even scarcer, with 21 and 4 samples, respectively. For 20 out of the 31 analyzed phyla, there was only one observed habitat, and in most of these cases, it was the marine habitat, except for Onychophora, where all samples were terrestrial. Habitat variability was remarkable in Arthropoda, Bryozoa, Mollusca, and Platyhelminthes.

### Read taxonomic classification into viral families

Overall, the proportion of the pre-processed subset of reads with significant similarity to any viral protein sequence was lower than 2% for approximately 92% of samples (Fig. 1B). On average, 1% of reads were assigned as viral. There is an outlying sample in terms of the viral proportion of the reads population, which corresponds to the flatworm *Rhynchomesostoma* sp. (9%). This was likely influenced by the fact that this was the only sample with less than a million reads, leading to a more inaccurate estimation of its transcriptome. When sorting the phyla based on their median viral proportion of reads, it can be observed that phyla such as Echinodermata, Mollusca, and Onychophora lie on the lower end of viral abundance, while samples of Bryozoa and Rotifera harbor a larger proportion of viral RNA.

Additionally, when examined by habitat, freshwater samples showed the highest proportion of viral reads on average (1.7%), while terrestrial samples had the lowest (0.8%). These results reflect two sides of the same coin, as the higher viral abundance in freshwater samples aligns with the dominance of bryozoans, platyhelminths, and rotifers seen above. In contrast, Onychophora, the phylum with the lowest viral abundance, is strictly terrestrial.

The proportion of viral RNA was found to be much more variable in another study focusing on the RNA viromes of several invertebrate phyla, ranging from 0.05% to 87% (4). However, in another study on the virome of insect species *Aedes albopictus*, it was determined that the fraction of eukaryotic viral reads, this time including rRNA reads, ranged from 0.2% to 1.8% (19). Another publication showed that the viral proportion of reads between samples of two other mosquito species was different both on average and in the range of observed values (18). A virus enrichment step was not included in the extraction protocol of any of these studies. Comparatively, the figures reported in the present study are lower and display less variation than what was reported in the literature. However, it is important to note that all of the mentioned studies used taxonomic annotation of contigs instead of reads, which may have had an effect on the results (65).

The majority of reads assigned to viral sequences were classified under families of eukaryotic viruses (52%), while phages (including here viruses of archaea) constituted a minority of the viral RNA in the analyzed samples, making up an average of 14% (Fig. 1C). A remarkable proportion of viral reads was mapped to sequences of undetermined viruses or virus species that are not yet classified to viral families in the NCBI taxonomy database. This fraction of unclassified viruses ranged between 1% (*Pocillopora damicornis*, Cnidaria) and 69% (*Cladolabes schmeltzii*, Echinodermata) of the whole virome. Among the deeper explored phyla, half of the samples of Ctenophora and Porifera have estimated viromes with less than 25% of unclassified viruses, while for Rotifera and Brachiopoda, half of their samples harbor viromes with approximately 40% or more relative abundance of viruses of uncertain family (see Fig. S1A at https://git.csic.es/sfelenalab/invertebrates-virome/-/tree/main/Supplementary%20files). When considering habitats, the median proportion of unclassified viruses is 32% for terrestrial and marine habitats, while it reached 38% for intertidal and freshwater samples.

Excluding the unclassified fraction of the virome, the phage proportion was highly variable, even within phyla and habitats: the lowest value was observed for the kynoryhinc *Echinoderes dujardinii* (0.4%), while the sample with the highest relative abundance of phage reads was a sea cucumber, *C. schmeltzii* (99.4%). However, the median phage proportion of the classified virome ranged between 11% and 12% for all three most sampled habitats (freshwater, marine, and terrestrial). For most phyla, the phage proportion of the virome showed a positively skewed distribution. The phylum with the highest proportion of phages was Porifera (43.3% on average), which goes in line with recent discoveries on the dominance of viruses of the order *Caudovirales* among the viral communities of sponges (21).

Out of the 134 viral families identified in the 417 samples, 90 were known to infect eukaryotic organisms. Twenty-eight of these families were found only in four or less samples, which represents less than 1% of the whole data set (see Table S2 at https://git.csic.es/sfelenalab/invertebrates-virome/-/tree/main/Supplementary%20files). In contrast, 13 viral families were identified in more than 95% of the samples, and the presence of reads classified to five of these families was consistent across all 417 samples, namely, *Flaviviridae*, *Marseilleviridae*, *Mimiviridae*, *Phycodnaviridae*, and *Retroviridae*.

The average relative abundance was very uneven among families: *Retroviridae* dominated the eukaryotic viromes, accounting for 34.7% of reads assigned to eukaryotic viruses, followed by *Mimiviridae* at 23.5% (see Fig. S1B and Table S3 at https://git.csic.es/sfelenalab/invertebrates-virome/-/tree/main/Supplementary%20files). Besides *Retroviridae*, the group of highly abundant viral families was dominated by nucleocytoplasmic large DNA viruses (NCLDV): altogether, *Iridoviridae*, *Marseilleviridae, Mimiviridae*, *Phycodnaviridae*, *Pithoviridae*, and *Poxviridae* constituted on average 41% of reads among the estimated eukaryotic viromes. However, proteins identified within these ubiquitous families often exhibited eukaryotic orthologs or xenologs. For instance, actin-like products were erroneously classified to *Retroviridae* in great numbers (CAA25063.1, P00544.1, QDH76138.1, and QDH76139.1). Furthermore, numerous sequences assigned to NCLDV families exhibited significant similarity to various host proteins, including heat-shock proteins, tRNA synthetase, histone, and ubiquitin sequences, among others. Although these genes with eukaryotic homologs can potentially be encoded by NCLDV (66–71), their abundance was significantly greater than those associated with the virus replication, which lack close homologs in the host genomes (72). This could hint in the direction of these hits representing host sequences in the samples, which could partly explain why a large part of the apparently more dominant viral families identified across the viromes were DNA viruses, contrary to the common consensus, which states that RNA viruses dominate invertebrate viromes (4, 5).

*Flaviviridae*, an RNA virus family known to infect invertebrates (73, 74), emerged as the third family with a higher average relative abundance of reads. However, its hits were often linked to a polyprotein of a bovine pestivirus species, incorporating a ubiquitin-like sequence (AAA42855.1). Flaviviruses are known to interact with host’s ubiquitin (75), and some flaviviruses of vertebrates even incorporate it into their genomes (76, 77). However, the scarcity of other matches for *Flaviviridae* and the recent incorporation of ubiquitin to genomes of flaviviruses make it unlikely that a significant number of these transcripts have a true viral origin. Consequently, the abundance of flaviviruses may have been notably overestimated within the viromes.

The surprising position of *Mitoviridae* as the fifth most consistently abundant viral family was found to be almost entirely caused by the identification of a highly conserved transcription elongation factor of eukaryotes that was misannotated as part of a mitovirus genome in the reference database (QDH87640.1).

Overall, it was challenging to determine whether the identified transcripts stemmed from genomes of independent viruses or from integrated virus sequences within the host’s genome because the study was based solely on RNA-Seq data. It is well known that endogenous viral elements (EVEs) of DNA and non-retroviral RNA viruses are widespread across invertebrate genomes, and these EVEs can be transcriptionally active (78, 79). Furthermore, EVEs of invertebrate genomes originate from a wider range of viral families when compared to vertebrate genomes, and the abundance and diversity of EVEs are highly variable even between closely related invertebrate species (80). Besides, in case of a true presence of retroviruses in the samples, it remains unknown if the viruses are found in the form of proviruses, as it has been reported to be common in insect retrovirus infections (81). It is also uncertain to which extent these integrated viruses could be transcriptionally active across the different invertebrate phyla.

With respect to the host of the detected viruses, 18 of the 30 most abundant viral families (see Table S3 at https://git.csic.es/sfelenalab/invertebrates-virome/-/tree/main/Supplementary%20files) have at least one member described that is able to infect invertebrates, considering the recent discoveries in *Hepeviridae* (82), *Mitoviridae* (83), and *Partitiviridae* (84). In many cases, the known host range of these families among invertebrates is limited to arthropods, but it is yet unknown if eventually they will be found in other invertebrate phyla. For instance, the presence of viruses of the family *Iridoviridae* was recently confirmed in sponges, thereby broadening its previously acknowledged host range (85). In our results, around 0.8% of all eukaryotic virus reads were mapped to *Iridoviridae* for Porifera samples on average, which is nonetheless lower than the mean relative abundance reported overall (see Table S3 at https://git.csic.es/sfelenalab/invertebrates-virome/-/tree/main/Supplementary%20files).

Sequences related to *Herpesviridae* were also identified in substantial amounts, following a trend seen in other studies on invertebrate viromes (23, 24, 86). In this case, most of the hits for *Herpesviridae* were mainly related to sequences of thymidylate synthase, which is encoded both by the genomes of herpesviruses and eukaryotes (87). To a lesser extent, hits to both subunits of ribonucleotide reductase were also frequent for *Herpesviridae*. This enzyme is also encoded by herpesviruses and their hosts and has a key role at different stages of the virus’s replication (88, 89). Although *Herpesviridae* is acknowledged to harbor exclusively vertebrate-infecting viruses, it has recently been suggested that the closely related family of *Malacoherpesviridae*, whose only two members are mollusk-infecting viruses, could be largely underexplored (90). In our study, sequences classified to *Malacoherpesviridae* were rare, absent from most of the samples and mostly related to a single hit, a putative ATP-dependent DNA ligase (YP_006908759). This protein is not encoded by viruses of *Herpesviridae*, but it has been annotated with this function in three different malacoherpesvirus genomes. Apparently, this protein shows especially close similarity to the ATP-dependent DNA ligase of invertebrates, mainly arthropods, nematodes, placozoans, and platyhelminths. BLASTp searches were performed for the query sequences that matched this protein, and significant similarities to the homologs within invertebrate genomes were found. Thus, it is unclear to which extent the abundance and ubiquitousness of herpes-like viruses were overestimated.

Establishing the hosts of viruses discovered through metatranscriptomics presents significant challenges, given the coexistence of multiple potential hosts within a single sample (15). Generally, the microbiome of an invertebrate constitutes a diverse set of organisms that can serve as hosts for very different viruses. Although the bacterial part of the microbiome can be ignored in this context, because phages have been excluded from the analysis, the eukaryotic fraction is nowhere near negligible in invertebrate microbiomes (91). The common occurrence of these symbioses could have further contributed to the reported abundance of protist-infecting NCLDVs. Additionally, RNA from eukaryotic viruses can be present in dietary components, ranging from other invertebrates to protists, algae, or plants, or they can be present simply due to sample contamination. Host determination lies beyond the scope of the current study, and host–virus associations are only tentatively inferred from the host ranges documented in the existing literature for each family.

The individual virome compositions for all the analyzed samples, as well as the aggregated viromes by phylum and habitat, can be accessed in an online resource (provisionally accessible at http://invertviromes.i2sysbio.uv.es/).

### Virome diversity and community structure analysis

**Fig 3:**
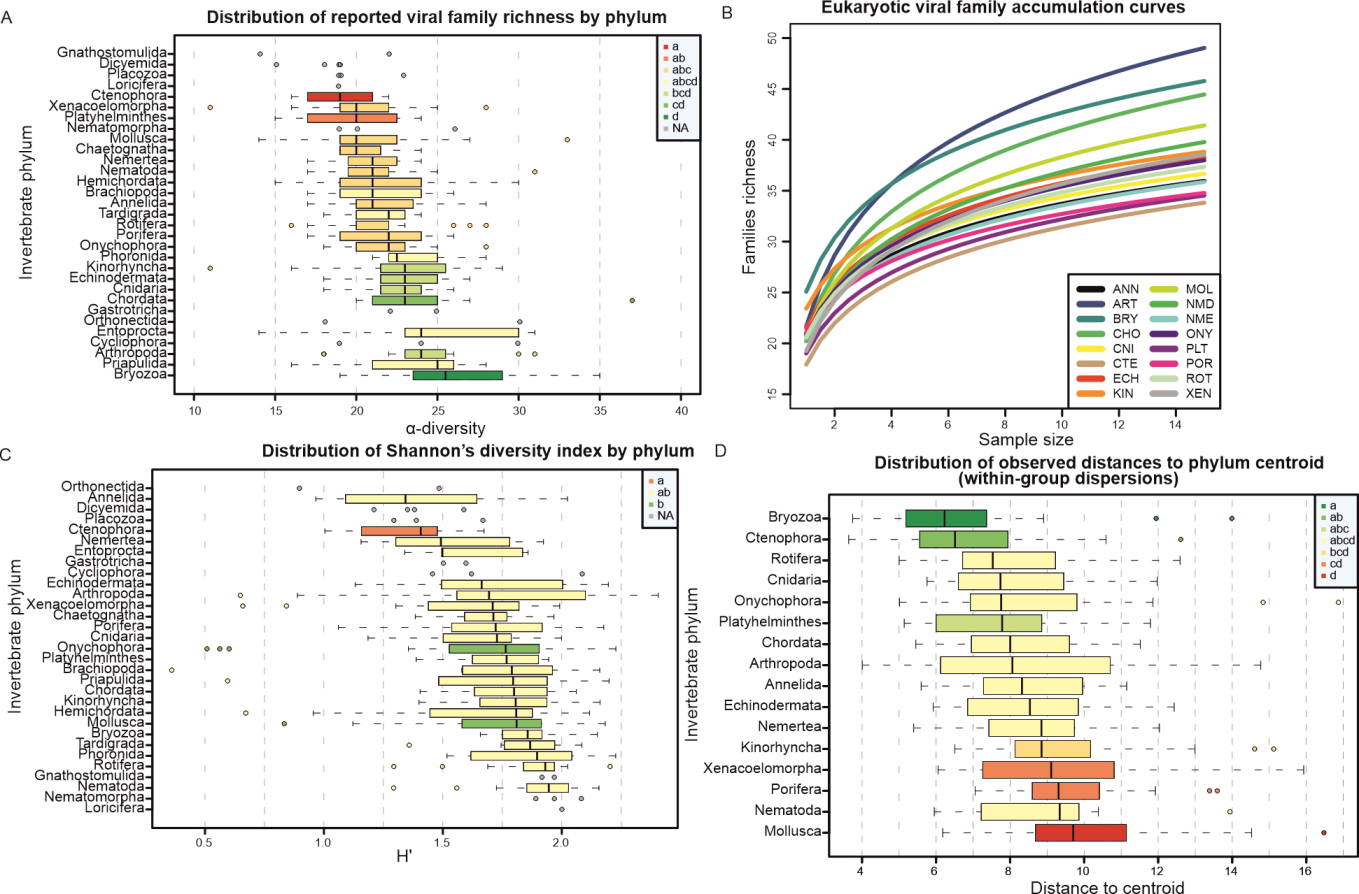
Comparison of the estimated virome diversity between phyla. (A) Observed distribution of the richness in eukaryotic viral families (α-diversity) for the samples of each phylum. Phyla represented by less than five samples were excluded from the ANOVA analysis comparing α-diversity between phyla. The legend displays the groups defined by the post hoc Tukey test. (B) Eukaryotic viral families accumulation curves by phylum. At each sample size, 50 iterations were performed to extract different samples of each size and to compute their α-diversity. A linear model was fit for the calculated values against the logarithm of the sample size to display the curves. Only phyla with 15 or more samples were included. Phylum abbreviations: Annelida (ANN), Arthropoda (ART), Bryozoa (BRY), Chordata (CHO), Cnidaria (CNI), Ctenophora (CTE), Echinodermata (ECH), Kinorhyncha (KIN), Mollusca (MOL), Nematoda (NMD), Nemertea (NME), Onychophora (ONY), Platyhelminthes (PLT), Porifera (POR), Rotifera (ROT), and Xenacoelomorpha (XEN). (C) Observed distribution of the Shannon’s diversity index for the eukaryotic fraction of the viromes. Phyla represented by less than five samples were excluded from the ANOVA analysis. A transformation of the family Box-Cox, a monotonic increasing function, was performed to avoid heteroscedasticity in the ANOVA model. The legend displays the groups defined by the post hoc Tukey test using the transformed values. (D) Distribution of the within-phylum variability of the virome compositions. The Euclidean distances of the *clr-*transformed compositions were obtained to compute the phylum centroid and the distances of each sample to its phylum centroid. Distances to phylum centroid were analyzed with an ANOVA model for the phyla with 15 or more samples. The legend displays the groups defined by the post hoc Tukey test.

On average, approximately 22 (interquartile range 19–24) different eukaryotic viral families were found for each sample. The most extreme cases were the sample of *Botryllus schlosseri* (Tunicata) with 37 identified families, and the samples of *Echinoderes dujardinii* (Kinorhyncha) and *Xenoturbella bocki* (Xenacoelomorpha) with only 11 families (Fig. 3A). Overall, α-diversity was similar between the samples, a trend congruent with findings from other studies on invertebrate viromes that employed a comparable RNA-Seq analysis pipeline (24).

The ANOVA model for family richness that involved both phylum and habitat as additive factors showed the best fit in terms of AIC. Notably, the influence of phylum on α-diversity differences was significantly more pronounced (*P* < 0.0001) compared to habitat (*P* ≈ 0.05). The significance of regression parameters for habitat groups became lower when phylum was added to the model, which supports the idea that a remarkable degree of collinearity exists between both factors, with phylum grouping being more informative. According to *R*^2^, 23% of the variance in family richness was explained by phylum and habitat groupings together. Homoscedasticity was checked for both factors (*P* > 0.05).

The rejection of the null hypothesis in the ANOVA model suggests that some differences in the average family richness exist between the phyla. Post hoc pairwise comparisons revealed that bryozoans showed a significantly higher family richness than the majority of tested phyla (Fig. 3A). In sharp contrast, Ctenophora was the phylum with the lowest number of different viral families per sample (Fig. 3A). The outlying sample of Chordata (Tunicata) shifted its mean to positive values compared to its median, which makes it the phylum with the second highest number of viral families on average. When seen by habitat, terrestrial and marine samples had an average α-diversity almost identical to the global mean. The average viral family richness for freshwater samples was slightly higher at 23 families, and intertidal zone samples showed the lowest α-diversity overall with 21 distinct families on average per virome. Compared to purely marine habitats, intertidal zone habitats pose a series of stresses to local organisms (92), which might reduce the range of hosts that viruses are adapted to and can find in intertidal zones. This could help to explain the observed small difference in α-diversity observed between habitats.

Family richness was also analyzed by phylum and habitat using rarefaction curves (Fig. 3B; see Fig. S2A at https://git.csic.es/sfelenalab/invertebrates-virome/-/tree/main/Supplementary%20files). This revealed that a number of samples >15 seem to be required to capture the full range of distinct viral families within any phylum, as none of the curves reached a plateau at this sample size. However, the lack of available samples for some phyla in the SRA database makes it impossible to reach that plateau. This should point out that more sequencing and explorative efforts are needed to describe the full diversity of invertebrate viruses.

In the case of Arthropoda, the accumulation curve seemed to be exceptionally steep, which suggests that the sampled transcriptomes for this phylum harbored the most diverse sets of viral families (Fig. 3B). Other phyla that stand out for a higher accumulation of viral families were Bryozoa, Chordata (Tunicata), and Mollusca. However, Bryozoa differed from the rest of the cited phyla by starting at a much higher value of α-diversity. On the lower end, Ctenophora, Platyhelminthes, and Porifera showed the lowest accumulation of viral families as the sample size increased (Fig. 3B). It does not seem that habitat affiliations are the main cause of these differences between phyla, because single habitat phyla were found on both extremes, just as phyla with more variable habitats. Interestingly, two of the phyla with the lowest rate of viral families accumulation are precisely the earliest branching animal phyla: Ctenophora and Porifera (93).

The observed Shannon’s *H′* values ranged between 0.4 and 2.4, lying in most cases between 1.5 and 2 (Fig. 3C). *H*′ was more variable than family richness, which suggests that evenness introduced a substantial degree of variation between the estimated viromes. Roughly 4% of the samples had viromes with *H* ′ < 1, which might have been caused by one or few viral families dominating the virome. This unevenness could be the consequence of ongoing infection, where transcripts associated with infecting viruses become much more abundant.

In this case, the best ANOVA model fit was the one-way model with phylum as the single covariate, which suggests that habitat was not as informative for the observed differences in *H′*. Because Levene’s test showed rejection of the hypothesis of homogeneity of variances across the phyla, a Box-Cox transformation was applied. Although the applied transformation could hinder the interpretation of the Tukey’s post hoc test, the use of the untransformed *H′* was ruled out to avoid misleading results. Mollusca and Onychophora appeared to have significantly higher mean in *H*′ when compared to Ctenophora (Fig. 3C). However, Mollusca and Onychophora did not show exceptionally high *H*′ values, it was their group sizes that were exceptionally large. Moreover, Ctenophora appears once again to have less diverse viromes also according to this measure (Fig. 3C).

All four network-level measures computed for the bipartite network of viral families, and invertebrate species were found to significantly differ from the estimates obtained from the null model (see Table S4 at https://git.csic.es/sfelenalab/invertebrates-virome/-/tree/main/Supplementary%20files). In particular, the observed linkage density, nestedness, and *Q* modularity were significantly higher than expected by chance, while the network-level specialization index *H*_2_′ was lower. In all cases, especially for *H*_2_′ and *Q*, the rejection of *z*-tests appears to be driven more by the high dimensionality of the matrix rather than substantial differences between observed measures and values estimated under the null model.

**Fig 4:**
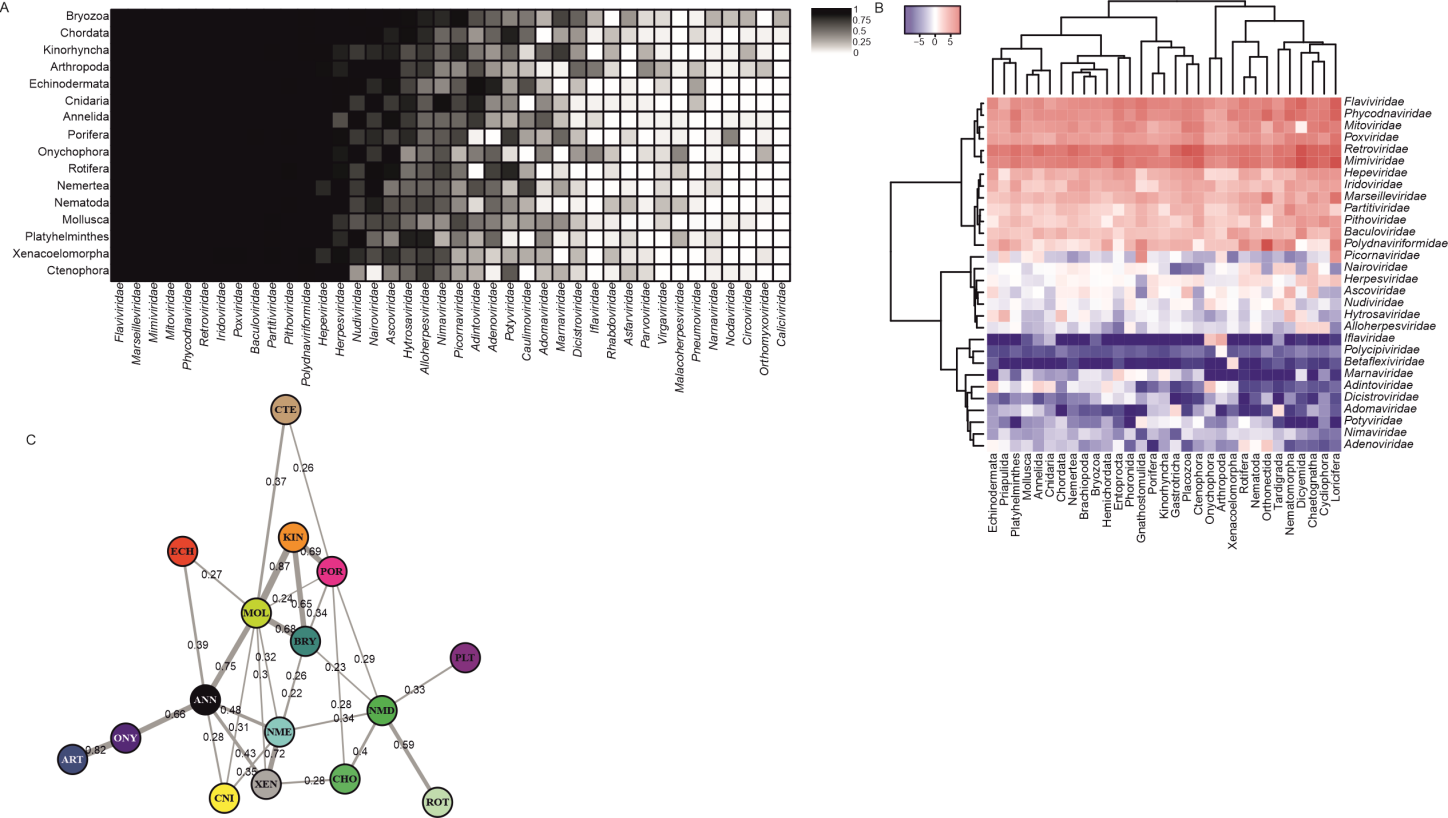
Compositional analysis of the viromes for phyla with 15 or more samples. (A) Virus-invertebrate interaction matrix by phylum. The color gradient illustrates the proportion of samples by phylum where each particular viral family was present. All 40 viral families shown are present in 20% of the samples of at least one phylum. Ordination of rows and columns highlights the nested nature of the interaction network. (B) Heatmap and hierarchical clustering analysis by phylum of the 30 most abundant eukaryotic viral families. Relative abundance vectors of viral families were aggregated by phylum, giving the same weight to each sample prior to performing the *clr* transformation. The hierarchical clustering of both phyla and viral families was performed using the Ward2 method. The legend displays the scale of the *clr* values. (C) Virome centroid equivalence network by phylum. Pairwise PERMANOVA and PERMDISP tests were computed in 1,500 bootstrap iterations for the *clr* coefficients vectors of each combination of phyla. Differences between phyla were considered significant only when PERMANOVA’s H_0_ was rejected but PERMDISP’s H_0_ was accepted. Links are shown connecting nodes only when the proportion of iterations, where differences between phyla were not significant, was higher than 0.2. Bootstrap support values are shown for each link. Phylum abbreviations: Annelida (ANN), Arthropoda (ART), Bryozoa (BRY), Chordata (CHO), Cnidaria (CNI), Ctenophora (CTE), Echinodermata (ECH), Kinorhyncha (KIN), Mollusca (MOL), Nematoda (NMD), Nemertea (NME), Onychophora (ONY), Platyhelminthes (PLT), Porifera (POR), Rotifera (ROT), and Xenacoelomorpha (XEN).

The high degree of nestedness in the network might suggest the coexistence of both generalist and specialist organisms on both sides of the matrix. This combination of high nestedness and low modularity is a recurring pattern observed in phage-bacteria and virus-plant networks (94, 95). As shown in Figure 4A, the representation of a bipartite matrix by phylum visually highlights nestedness, mainly through the aforementioned group of ubiquitous viral families in contrast to rarer families. This set of ubiquitous families, comprising *Baculo-*, *Flavi-*, *Partiti-*, *Polydna-*, *Retroviridae*, and several NCLDV families, could form a core virome that is common to the majority of the analyzed samples. Nevertheless, nestedness could have been overestimated because of the erroneous classification of highly conserved host sequences as viral, as seen in some of these families.

Specialization indices (*d*′) were calculated for both invertebrate species and viral families within the bipartite matrix. Remarkably, the distribution of *d′* values was almost identical for both viruses and hosts, both having a mean *d*′ = 0.01. The highest observed *d′* values did not surpass 0.06 in any case. These low *d′* values suggest that interactions are not restricted to specific pairs of organisms. Consequently, any of the 30 viral families included in this matrix could potentially be found in any of the viromes, as determined by the taxonomic classification pipeline employed. Furthermore, a comparison of *d*′ indices among phyla using an ANOVA model revealed a significant association between phylum grouping and variations in *d*′ values (*P* < 0.0001). Notably, species belonging to Arthropoda, Mollusca, Onychophora, and Tardigrada displayed a higher degree of specialization in hosting viral families. In contrast, Bryozoa, Chaetognatha, and Nematoda were characterized by a greater tendency toward generalism of viral families.

### Virome composition analysis

In order to assess the dominance of each viral family within the viromes, the matrix of relative abundances was transformed into *clr* coefficients. Viral families with an average relative abundance below 0.01% were excluded. The transformed matrix was used to examine viral family dominance across phyla through a heatmap (Fig. 4B). Principal component analysis was also performed, but the biplot representation of the results was not visually intuitive because of the large amount of data points and different groups defined by phylum and habitat in this data set. The first two principal components accounted for 23% of the total variance, indicating a limited correlation among the *clr* coefficients of the 30 viral families studied.

Hierarchical clustering analysis revealed the presence of three clusters of viral families that were distinguishable with considerable resolution (Fig. 4B). The first cluster consisted of 13 superabundant, ubiquitous viral families (*Baculo*-, *Flavi*-, *Hepe*-, *Irido*-, *Marseille*-, *Mimi*-, *Mito*-, *Partiti*-, *Phycodna*-, *Pitho*-, *Polydnaviriformi*-, *Pox-*, and *Retroviridae*) showing positive *clr* coefficients for all phyla (Fig. 4B). Within this cluster, eight of the families harbored viruses with dsDNA genomes (*Baculo*-, *Irido*-, *Marseille*-, *Mimi*-, *Phycodna*-, *Pitho*-, *Polydnaviriformi*-, and *Poxviridae*), with a notable presence of NCLDV families. In the analysis, all NCLDV families except *Ascoviridae* clustered together in this group. Furthermore, this cluster was the one with the highest distance to the other two clusters.

A second cluster contained less abundant families with values of *clr* coefficients oscillating around zero (*Alloherpes*-, *Asco*-, *Nairo*-, *Herpes*-, *Hytrosa-*, *Nudi*-, and *Picornaviridae*), signifying their dominance within the virome only for specific phyla (Fig. 4B). DNA virus families are again more frequent here (five out of seven), and the acknowledged host ranges of these families include invertebrates in all cases except for *Alloherpes-* and *Herpesviridae*.

The last cluster is constituted by even rarer viral families that were only found to be dominant in the viromes of one or very few phyla: *Adeno*-, *Adinto*-, *Adoma*-, *Betaflexi*-, *Dicistro*-, *Iflavi*-, *Marna*-, *Nima*-, *Polycipi*-, and *Potyviridae* (Fig. 4B). Among the 10 viral families included in this cluster, six of them were families of RNA viruses. The two only families of exclusively plant and/or fungi-infecting viruses of this 30 families’ data set were found in this cluster: *Betaflexi-* and *Potyviridae*. In both cases, sequences similar to these families were identified predominantly in phyla of marine invertebrates, where the occurrence of these viruses has not been confirmed. In the case of *Betaflexiviridae*, most hits pointed to the occurrence of viral replicases and RNA-dependent RNA polymerases within the samples. Meanwhile, most of the hits of *Potyviridae* were related to an inosine triphosphate pyrophosphatase (ITPase) protein, which is only present in two potyvirus species (96). This protein shows significant sequence homology to ITPase proteins of plants, and, to a lesser extent, in invertebrates. However, hits to coat proteins of potyviruses were also reported in several samples. True viral presence of both families among the samples seems likely, although the abundance of potyviruses was probably overestimated. Interestingly, *Betaflexiviridae*-like sequences were also identified in different transcriptomes of marine mollusk samples by a recent study (97).

Unlike the clustering observed for viral families, the separation of phyla into clusters was less obvious. Interpreting the clusters proved more challenging, not only because of the clustering’s low resolution, but also for the apparent lack of consistent patterns within the clusters based on the phylogenetic or habitat affinities between the phyla. One of the groups with a greater distance to the rest was the one formed by Arthropoda and Onychophora, the two phyla that are closely related phylogenetically (98). This suggests that their viromes have similar compositions at the family level. For instance, these two were the only phyla where sequences of *Iflaviridae*, a family of insect-infecting RNA viruses, were found with a high relative abundance.

PERMANOVA models were fitted in order to investigate the potential impact that the phylum and the habitat of an invertebrate sample could have on the composition of its virome. The data set was restricted to phyla and habitats with 15 or more samples for the analysis. In both one-way models, it was seen that virome compositions were significantly influenced by the host’s phylum and habitat (*P* < 0.05). Nonetheless, the effect of the host’s phylum on the compositions was nearly six times greater than that of habitat (see Table S5 at https://git.csic.es/sfelenalab/invertebrates-virome/-/tree/main/Supplementary%20files). It has to be taken into account that the habitat effect could have been underestimated in this study because of the lack of a more comprehensive set of habitat categories. The phylum factor alone accounted for approximately 26% of the variance in the Euclidean distances matrix.

In the additive two-way model, regression parameters associated to both factors remained significant, suggesting that collinearity, although present, does not totally neglect the separate effect of both factors in shaping the viromes. Furthermore, the model considering phylum and habitat interaction showed that even the interaction had significant regression parameters. Nevertheless, the slight improvement in goodness of fit did not offset the increase in model complexity of the two-way models, because the model with phylum as its single covariate was the one that minimized AIC. It was checked that similar results overall were obtained when using the Bray-Curtis dissimilarities of the raw relative abundances matrix, which is a more traditional approach.

However, it is important to note that the rejection of the null hypothesis in PERMANOVA could also arise from varying within-group dispersions in the case of unbalanced designs (43). The result of PERMDISP showed that within-group dispersions were indeed heterogeneous between phyla (*P* < 0.05). Virome composition variation was more thoroughly examined using a procedure based on the analysis of the distances to the centroid, i.e., the “average virome,” in each phylum. The strategy of fitting an ANOVA model and performing a Tukey’s post hoc test was also used for this variable and showed that distances to centroid were significantly different between some phyla (*P* < 0.05). Interestingly, most of the phyla exhibiting extreme distances to centroid have already been mentioned for having an exceptional behavior in terms of α-diversity. Bryozoans appeared to have the most consistently similar virome compositions, closely followed by ctenophores. Conversely, mollusks showed the greatest mean distance to the centroid, together with xenacoelomorphs and sponges (Fig. 3D).

The habitat variability between phyla does not appear to be the primary driver of these observed differences, as phyla with both diverse and singular habitats were represented at both extremes. One potential influencing factor for these findings could be the phylogenetic proximity among species within the same phylum. This study treats species as independent observations of their respective phylum, disregarding their membership in the same genus, family, or order. This is a limitation of this study, because the phylogenetic constraints underlying species relatedness within phyla were not taken into consideration.

Additionally, the viromes of Arthropoda, Onychophora, and Xenacoelomorpha exhibit notable variability in their distances to the phylum centroid. This indicates that within these phyla, certain samples are positioned very close to the centroid, while others are located considerably farther away (Fig. 3D). That is to say, these phyla tend to show a high variability in their virome compositions, not only as the different viral families that can be found in these viromes, but also in what relative abundances they are found.

A pairwise algorithm with bootstrap resampling and simultaneous PERMANOVA and PERMDISP testing was implemented to further investigate if virome compositions were different between phyla. To ensure balanced group sizes, the sample count per phylum was standardized to 15. The results highlighted that a significant majority of phyla pairs exhibited dissimilar centroid locations in most iterations (see Fig. S2B at https://git.csic.es/sfelenalab/invertebrates-virome/-/tree/main/Supplementary%20files), even if a *P* value adjustment method was employed to correct for multiple pairwise comparisons. Moreover, equivalence of centroid locations was observed as the most frequent result through the iterations only for a limited number of phyla pairs. Surprisingly, heterogeneous within-group dispersions were not found to be the most frequent result in any case, in contrast to earlier findings using the whole data set.

The results of this matrix were condensed into a network to allow a visual interpretation of the results (Fig. 4C). Curiously, all phyla remained connected to at least some other phylum, meaning that their virome composition centroids were not considered significantly different to each other. This resulted in a continuous network, where no phylum had an outlying virome composition that differed from the rest. However, there were some cases of phyla with few and weak connections, such as Ctenophora, Echinodermata, and Platyhelminthes. In contrast, Mollusca was the phylum with the highest number of connections, some of which were strong, meaning that the “average virome” of mollusks was not distinguishable from that of some other phyla.

Some of the strongest similarities between virome compositions that can be observed in the network can also be identified in the heatmap (Fig. 4B), namely, Arthropoda-Onychophora, Mollusca-Annelida, Kinorhyncha-Porifera, and Rotifera-Nematoda. Viromes of Arthropoda only appear to be closely similar to those of Onychophora, which also shares a rather pronounced centroid homology with Annelida, all three being the phyla dominated by terrestrial species. In the case of Arthropoda and Onychophora, both belong to the clade of Panarthropoda, alongside Tardigrada (Fig. 1C) (63), which could explain the remarkable degree of similarity observed between their virome compositions. Unfortunately, Tardigrada could not be included in this part of the analysis to test this hypothesis further because of its low sample count. Likewise, the reported similarity between the viromes of Annelida and Mollusca could be attributed to their phylogenetic relatedness, since both group within a subclade under the superphylum Lophotrochozoa (Fig. 1C) (99). Moreover, the average viromes of Nemertea showed a weak resemblance to those of the rest of the phyla in this subclade, while the similarity between the viromes of Bryozoa and Mollusca, other phyla of this subclade, was one of the highest (Fig. 4C). The hypothesis behind this phenomenon of closely related phyla having equivalent viromes is that viromes may have coevolved with their hosts. The viromes of these phyla are probably less influenced by changes in the host’s habitat or lifestyle during evolution. Nonetheless, this explanation seems applicable only for the virome composition similarities of a limited number of phyla.

In other cases, such as the reported similarity of virome compositions between Kinorhyncha and Porifera or Nemertea and Xenacoelomorpha, the explanation could not be found in the phylogenetic relationships between the phyla. Rather, these phyla appeared to have similar habitats among their samples. However, it is unknown why the viromes of these specific phyla were found to be so similar, given that many other phyla have apparently similar habitats. For instance, it has been described that kinorhynchs can be found inhabiting sponges, although this is not thought to be the norm and they are generally interstitial organisms (100, 101). Therefore, a more comprehensive analysis of lifestyles, diet, and associations with other organisms would be needed in this regard.

The reason behind some other phyla having notable similarities across their average virome compositions, such as Kinorhyncha and Mollusca, Bryozoa and Kinorhyncha, or Nematoda and Rotifera, remains uncertain, because these specific pairs of phyla do not exhibit any close phylogenetic or ecological affinity. However, in summary, our findings align with the results of Shi et al. (4). They underscore that the assumption of virus–host co-divergence cannot be inferred from all cases where viromes exhibit similarities across different phyla, because transmission of viruses between divergent host taxa has been frequent throughout the evolutionary history of viruses (102).

### Conclusions

A taxonomic classification pipeline for RNA and DNA viruses was applied to more than 400 RNA-Seq data sets of invertebrates comprising 31 phyla. Overall, the estimated viromes were not widely variable in terms of α-diversity, although significant differences in the means could be found between the phyla. Additionally, viromes were consistently dominated by a set of ubiquitous families, the abundance of which was likely overestimated due to the misclassification of host sequences as viral, as such is the case for families like *Retroviridae*, *Flaviviridae*, or *Herpesviridae*. The structure of the invertebrate-virus bipartite network showed that its interactions were highly nested, which hints to a shared core virome across the animal tree of life. In the majority of the most abundant viral families found, their acknowledged host ranges in the literature included invertebrates. However, the confirmed host ranges of these families are limited to overrepresented phyla such as Arthropoda or Mollusca, and the presence of most of these families has still to be confirmed for the most understudied phyla. Consequently, virus sequences in the reference databases are also biased toward these relevant phyla.

Further improvements in the resolution of the animal tree of life could help improve the interpretation of invertebrate viromes, as many nodes remain unresolved and changes in the phylogeny of animal phyla keep occurring to this date. However, evidence shows that the host’s phylum has a predominant role in shaping its virome composition, rather than its habitat. A main limitation for this observation could be the effect that differential expression of host genes that had been erroneously classified as viral could have had between the phyla. Also, it is important to note that the reconstruction of the host’s habitat was based on the taxonomy of the host rather than the sampling source, due to the lack of metadata in many cases. Consequently, the host’s habitat affiliation was limited to a very generic set of habitat categories.

As a concluding remark, comparability to other studies has remained limited due to the lack of studies analyzing together the RNA and DNA viromes of invertebrates at the phylum level. Additionally, ecological community structure of invertebrate-virus interactions at the phylum and family levels, respectively, and the systematic review of similarities between the virome compositions of the different phyla had not yet been published. Finally, we recommend that similar studies implement a more intensive filtering of host sequences that is not limited to rRNA transcripts, although a trade-off will always exist between false positives and false negatives when classifying host sequences as viral and when excluding transcripts encoded by the viral genome with true homology to eukaryotic genes, respectively.

## Data Availability

Supplemental files can be downloaded from https://git.csic.es/sfelenalab/invertebrates-virome/-/tree/main/Supplementary%20files. All scripts used for the selection of samples, the read taxonomic classification pipeline, and the statistical analysis, as well as the generation of figures presented in this report, can be found at https://git.csic.es/sfelenalab/invertebrates-virome under the directory “Data and Scripts.” Intermediate data sets, in addition to the main data table in CSV format, can be found alongside the scripts. The code for the Shiny app is also available at the same Git repository under the directory “Shiny app,” allowing the app to be run locally.

## References

[1] Breitbart M, Rohwer F. 2005. Here a virus, there a virus, everywhere the same virus?. Trends Microbiol 13:278–284. doi:10.1016/j.tim.2005.04.003 15936660

[2] Koonin EV, Senkevich TG, Dolja VV. 2006. The ancient virus world and evolution of cells. Biol Direct 1:29. doi:10.1186/1745-6150-1-29 16984643 PMC1594570

[3] Wasik BR, Turner PE. 2013. On the biological success of viruses. Annu Rev Microbiol 67:519–541. doi:10.1146/annurev-micro-090110-102833 23808330

[4] Shi M, Lin XD, Tian JH, Chen LJ, Chen X, Li CX, Qin XC, Li J, Cao JP, Eden JS, Buchmann J, Wang W, Xu J, Holmes EC, Zhang YZ. 2016. Redefining the invertebrate RNA virosphere. Nature 540:539–543. doi:10.1038/nature20167 27880757

[5] Porter AF, Shi M, Eden JS, Zhang YZ, Holmes EC. 2019. Diversity and evolution of novel invertebrate DNA viruses revealed by meta-transcriptomics. Viruses 11:1092. doi:10.3390/v11121092 31775324 PMC6950620

[6] Starr EP, Nuccio EE, Pett-Ridge J, Banfield JF, Firestone MK. 2019. Metatranscriptomic reconstruction reveals RNA viruses with the potential to shape carbon cycling in soil. Proc Natl Acad Sci USA 116:25900–25908. doi:10.1073/pnas.1908291116 31772013 PMC6926006

[7] Wolf YI, Silas S, Wang Y, Wu S, Bocek M, Kazlauskas D, Krupovic M, Fire A, Dolja VV, Koonin EV. 2020. Doubling of the known set of RNA viruses by metagenomic analysis of an aquatic virome. Nat Microbiol 5:1262–1270. doi:10.1038/s41564-020-0755-4 32690954 PMC7508674

[8] Wu H, Pang R, Cheng T, Xue L, Zeng H, Lei T, Chen M, Wu S, Ding Y, Zhang J, Shi M, Wu Q. 2020. Abundant and diverse RNA viruses in insects revealed by RNA-Seq analysis: ecological and evolutionary implications. mSystems 5:e00039-20. doi:10.1128/mSystems.00039-20 32636338 PMC7343303

[9] Neri U, Wolf YI, Roux S, Camargo AP, Lee B, Kazlauskas D, Chen IM, Ivanova N, Zeigler Allen L, Paez-Espino D, et al.. 2022. Expansion of the global RNA virome reveals diverse clades of bacteriophages. Cell 185:4023–4037. doi:10.1016/j.cell.2022.08.023 36174579

[10] Zayed AA, Wainaina JM, Dominguez-Huerta G, Pelletier E, Guo J, Mohssen M, Tian F, Pratama AA, Bolduc B, Zablocki O, et al.. 2022. Cryptic and abundant marine viruses at the evolutionary origins of earth's RNA virome. Science 376:156–162. doi:10.1126/science.abm5847 35389782 PMC10990476

[11] Simmonds P, Adams MJ, Benkő M, Breitbart M, Brister JR, Carstens EB, Davison AJ, Delwart E, Gorbalenya AE, Harrach B, et al.. 2017. Consensus statement: virus taxonomy in the age of metagenomics. Nat Rev Microbiol 15:161–168. doi:10.1038/nrmicro.2016.177 28134265

[12] Simmonds P, Adriaenssens EM, Zerbini FM, Abrescia NGA, Aiewsakun P, Alfenas-Zerbini P, Bao Y, Barylski J, Drosten C, Duffy S, et al.. 2023. Four principles to establish a universal virus taxonomy. PLoS Biol. 21:e3001922. doi:10.1371/journal.pbio.3001922 36780432 PMC9925010

[13] Zhang YZ, Shi M, Holmes EC. 2018. Using Metagenomics to characterize an expanding virosphere. Cell 172:1168–1172. doi:10.1016/j.cell.2018.02.043 29522738

[14] Harvey E, Holmes EC. 2022. Diversity and evolution of the animal virome. Nat Rev Microbiol 20:321–334. doi:10.1038/s41579-021-00665-x 34983966

[15] Cobbin JC, Charon J, Harvey E, Holmes EC, Mahar JE. 2021. Current challenges to virus discovery by meta-transcriptomics. Curr Opin Virol 51:48–55. doi:10.1016/j.coviro.2021.09.007 34592710

[16] Meng F, Ding M, Tan Z, Zhao Z, Xu L, Wu J, He B, Tu C. 2019. Virome analysis of tick-borne viruses in Heilongjiang province, China. Ticks Tick Borne Dis 10:412–420. doi:10.1016/j.ttbdis.2018.12.002 30583876

[17] Kondo H, Fujita M, Hisano H, Hyodo K, Andika IB, Suzuki N. 2020. Virome analysis of aphid populations that infest the barley field: the discovery of two novel groups of nege/kita-like viruses and other novel RNA viruses. Front Microbiol 11:509. doi:10.3389/fmicb.2020.00509 32318034 PMC7154061

[18] Shi C, Beller L, Deboutte W, Yinda KC, Delang L, Vega-Rúa A, Failloux AB, Matthijnssens J. 2019. Stable distinct core eukaryotic viromes in different mosquito species from guadeloupe, using single mosquito viral metagenomics. Microbiome 7:121. doi:10.1186/s40168-019-0734-2 31462331 PMC6714450

[19] Shi C, Zhao L, Atoni E, Zeng W, Hu X, Matthijnssens J, Yuan Z, Xia H. 2020. Stability of the virome in lab- and field-collected Aedes albopictus mosquitoes across different developmental stages and possible core viruses in the publicly available virome data of Aedes mosquitoes. mSystems 5:e00640-20. doi:10.1128/mSystems.00640-20 32994288 PMC7527137

[20] Huang HJ, Ye ZX, Wang X, Yan XT, Zhang Y, He YJ, Qi YH, Zhang XD, Zhuo JC, Lu G, Lu JB, Mao QZ, Sun ZT, Yan F, Chen JP, Zhang CX, Li JM. 2021. Diversity and infectivity of the RNA virome among different cryptic species of an agriculturally important insect vector: whitefly Bemisia tabaci. NPJ Biofilms Microbiomes 7:43. doi:10.1038/s41522-021-00216-5 33986295 PMC8119434

[21] Pascelli C, Laffy PW, Botté E, Kupresanin M, Rattei T, Lurgi M, Ravasi T, Webster NS. 2020. Viral ecogenomics across the porifera. Microbiome 8:144. doi:10.1186/s40168-020-00919-5 33008461 PMC7532657

[22] García-Bonilla E, Chaves-Moreno D, Riaño-Pachón D, Terán W, Acosta A, Junca H. 2021. The hologenome of Haliclona fulva (Porifera, demospongiae) reveals an abundant and diverse viral community. Front Mar Sci 8:1632. doi:10.3389/fmars.2021.736817

[23] Grasis JA, Lachnit T, Anton-Erxleben F, Lim YW, Schmieder R, Fraune S, Franzenburg S, Insua S, Machado G, Haynes M, Little M, Kimble R, Rosenstiel P, Rohwer FL, Bosch TCG. 2014. Species-specific viromes in the ancestral holobiont Hydra. PLoS One 9:e109952. doi:10.1371/journal.pone.0109952 25343582 PMC4208763

[24] Brüwer JD, Voolstra CR. 2018. First insight into the viral community of the cnidarian model metaorganism aiptasia using RNA-Seq data. PeerJ 6:e4449. doi:10.7717/peerj.4449 29507840 PMC5835348

[25] Lewandowska M, Hazan Y, Moran Y. 2020. Initial virome characterization of the common cnidarian lab model Nematostella vectensis. Viruses 12:218. doi:10.3390/v12020218 32075325 PMC7077227

[26] Vieira P, Subbotin SA, Alkharouf N, Eisenback J, Nemchinov LG. 2022. Expanding the RNA virome of nematodes and other soil-inhabiting organisms. Virus Evol 8:veac019. doi:10.1093/ve/veac019 35371560 PMC8967085

[27] Hewson I, Aquino CA, DeRito CM. 2020. Virome variation during sea star wasting disease progression in Pisaster ochraceus (Asteroidea, echinodermata). Viruses 12:1332. doi:10.3390/v12111332 33233680 PMC7699681

[28] Gudenkauf BM, Hewson I. 2016. Comparative metagenomics of viral assemblages inhabiting four phyla of marine invertebrates. Front Mar Sci 3:23. doi:10.3389/fmars.2016.00023

[29] Dunn CW, Giribet G, Edgecombe GD, Hejnol A. 2014. Animal phylogeny and its evolutionary implications. Annu Rev Ecol Evol Syst 45:371–395. doi:10.1146/annurev-ecolsys-120213-091627

[30] Olendraite I, Brown K, Firth AE. 2023. Identification of RNA virus-derived RdRp sequences in publicly available transcriptomic data sets. Mol Biol Evol 40:msad060. doi:10.1093/molbev/msad060 37014783 PMC10101049

[31] Perez-Riverol Y, Zorin A, Dass G, Vu MT, Xu P, Glont M, Vizcaíno JA, Jarnuczak AF, Petryszak R, Ping P, Hermjakob H. 2019. Quantifying the impact of public omics data. Nat Commun 10:3512. doi:10.1038/s41467-019-11461-w 31383865 PMC6683138

[32] Ahyong S, et al.. 2023. World register of marine species. Available from: https://www.marinespecies.org

[33] Kopylova E, Noé L, Touzet H. 2012. SortMeRNA: fast and accurate filtering of ribosomal RNAs in metatranscriptomic data. Bioinformatics 28:3211–3217. doi:10.1093/bioinformatics/bts611 23071270

[34] Bolger AM, Lohse M, Usadel B. 2014. Trimmomatic: a flexible trimmer for Illumina sequence data. Bioinformatics 30:2114–2120. doi:10.1093/bioinformatics/btu170 24695404 PMC4103590

[35] Menzel P, Ng KL, Krogh A. 2016. Fast and sensitive taxonomic classification for metagenomics with Kaiju. Nat Commun 7:11257. doi:10.1038/ncomms11257 27071849 PMC4833860

[36] Blüthgen N, Menzel F, Blüthgen N. 2006. Measuring specialization in species interaction networks. BMC Ecol 6:9. doi:10.1186/1472-6785-6-9 16907983 PMC1570337

[37] Almeida‐Neto M, Guimarães P, Guimarães PR Jr, Loyola RD, Ulrich W. 2008. A consistent metric for Nestedness analysis in ecological systems: reconciling concept and measurement. Oikos 117:1227–1239. doi:10.1111/j.0030-1299.2008.16644.x

[38] Newman MEJ. 2006. Modularity and community structure in networks. Proc Natl Acad Sci USA 103:8577–8582. doi:10.1073/pnas.0601602103 16723398 PMC1482622

[39] Gloor GB, Macklaim JM, Pawlowsky-Glahn V, Egozcue JJ. 2017. Microbiome datasets are compositional: and this is not optional. Front Microbiol 8:2224. doi:10.3389/fmicb.2017.02224 29187837 PMC5695134

[40] Xia Y, Sun J, Chen DG. 2018. Compositional analysis of microbiome data. In Statistical analysis of microbiome data with R. ICSA book series in statistics. Springer, Singapore.

[41] Anderson MJ. 2001. A new method for non-parametric multivariate analysis of variance. Austral Ecol 26:32–46. doi:10.1111/j.1442-9993.2001.01070.pp.x

[42] Anderson MJ. 2006. Distance-based tests for homogeneity of multivariate dispersions. Biometrics 62:245–253. doi:10.1111/j.1541-0420.2005.00440.x 16542252

[43] Anderson MJ, Walsh DCI. 2013. PERMANOVA, ANOSIM, and the mantel test in the face of heterogeneous dispersions: what null hypothesis are you testing?. Ecological Monographs 83:557–574. doi:10.1890/12-2010.1

[44] Fox J, Weisberg S. 2019. An R companion to applied regression. 3rd ed. Thousand Oaks,CA: Sage. https://socialsciences.mcmaster.ca/jfox/Books/Companion/.

[45] Graves S, Piepho H-P, Selzer L, Dorai-Raj S. 2023. multcompView: visualizations of paired comparisons (version 0.1-9) [software manual]. https://CRAN.R-project.org/package=multcompView.

[46] Venables WN, Ripley BD. 2002. Modern applied Statistics with S. 4th ed. Springer, New York. https://www.stats.ox.ac.uk/pub/MASS4/.

[47] Dormann CF, Frund J, Bluthgen N, Gruber B. 2009. Indices, graphs and null models: analyzing bipartite ecological networks. Open J Ecol 2:7–24. doi:10.2174/1874213000902010007

[48] Palarea-Albaladejo J, Martín-Fernández JA. 2015. zCompositions — R package for multivariate imputation of left-censored data under a compositional approach. Chemom Intell Lab Syst 143:85–96. doi:10.1016/j.chemolab.2015.02.019

[49] van den Boogaart KG, Tolosana-Delgado R. 2008. ”compositions”: a unified r package to analyze compositional data. Comput Geosci 34:320–338. doi:10.1016/j.cageo.2006.11.017

[50] Oksanen J, et al.. 2022. vegan: community ecology package (version 2.6-4) [software manual]. https://CRAN.R-project. org/package=vegan.

[51] Wickham H. 2016. ggplot2: elegant graphics for data analysis. Springer-Verlag New York.

[52] Zhao S, Guo Y, Sheng Q, Shyr Y. 2014. Heatmap3: an improved Heatmap package with more powerful and convenient features. BMC Bioinformatics 15:16. doi:10.1186/1471-2105-15-S10-P1624428894

[53] Csardi G, Nepusz T. 2006. The igraph software package for complex network research. InterJ, Comp Sys:1695.

[54] Zhang ZQ. 2013. Animal biodiversity: an outline of higher-level classification and survey of taxonomic richness (addenda 2013). Zootaxa 3703:1–82. doi:10.11646/zootaxa.3703.1.1 26146682

[55] Laumer CE, Fernández R, Lemer S, Combosch D, Kocot KM, Riesgo A, Andrade SCS, Sterrer W, Sørensen MV, Giribet G. 2019. Revisiting Metazoan phylogeny with genomic sampling of all phyla. Proc Biol Sci 286:20190831. doi:10.1098/rspb.2019.0831 31288696 PMC6650721

[56] Yamasaki H, Fujimoto S, Miyazaki K. 2015. Phylogenetic position of loricifera inferred from nearly complete 18S and 28S rRNA gene sequences. Zoological Lett 1:18. doi:10.1186/s40851-015-0017-0 26605063 PMC4657359

[57] Lu TM, Kanda M, Satoh N, Furuya H. 2017. The phylogenetic position of dicyemid mesozoans offers insights into spiralian evolution. Zoological Lett 3:6. doi:10.1186/s40851-017-0068-5 28560048 PMC5447306

[58] Laumer CE, Gruber-Vodicka H, Hadfield MG, Pearse VB, Riesgo A, Marioni JC, Giribet G. 2018. Support for a clade of placozoa and cnidaria in genes with minimal compositional bias. Elife 7:e36278. doi:10.7554/eLife.36278 30373720 PMC6277202

[59] Marlétaz F, Peijnenburg K, Goto T, Satoh N, Rokhsar DS. 2019. A new spiralian phylogeny places the enigmatic arrow worms among gnathiferans. Curr Biol 29:312–318. doi:10.1016/j.cub.2018.11.042 30639106

[60] Herranz M, Stiller J, Worsaae K, Sørensen MV. 2022. Phylogenomic analyses of mud dragons (Kinorhyncha). Mol Phylogenet Evol 168:107375. doi:10.1016/j.ympev.2021.107375 34952205

[61] Juravel K, Porras L, Höhna S, Pisani D, Wörheide G. 2023. Exploring genome gene content and morphological analysis to test recalcitrant nodes in the animal phylogeny. PLoS One 18:e0282444. doi:10.1371/journal.pone.0282444 36952565 PMC10035847

[62] Schultz DT, Haddock SHD, Bredeson JV, Green RE, Simakov O, Rokhsar DS. 2023. Ancient gene linkages support ctenophores as sister to other animals. Nature 618:110–117. doi:10.1038/s41586-023-05936-6 37198475 PMC10232365

[63] Wu R, Pisani D, Donoghue PCJ. 2023. The unbearable uncertainty of panarthropod relationships. Biol Lett 19:20220497. doi:10.1098/rsbl.2022.0497 36628953 PMC9832341

[64] Zhang Y-Y, Chen Y, Wei X, Cui J. 2022. Viromes in marine ecosystems reveal remarkable Invertebrate RNA virus diversity. Sci China Life Sci 65:426–437. doi:10.1007/s11427-020-1936-2 34156600

[65] Tamames J, Cobo-Simón M, Puente-Sánchez F. 2019. Assessing the performance of different approaches for functional and taxonomic annotation of metagenomes. BMC Genomics 20:960. doi:10.1186/s12864-019-6289-6 31823721 PMC6902526

[66] Raoult D, Audic S, Robert C, Abergel C, Renesto P, Ogata H, La Scola B, Suzan M, Claverie J-M. 2004. The 1.2-megabase genome sequence of mimivirus. Science 306:1344–1350. doi:10.1126/science.1101485 15486256

[67] Boyer M, Yutin N, Pagnier I, Barrassi L, Fournous G, Espinosa L, Robert C, Azza S, Sun S, Rossmann MG, Suzan-Monti M, La Scola B, Koonin EV, Raoult D. 2009. Giant marseillevirus highlights the role of amoebae as a melting pot in emergence of chimeric microorganisms. Proc Natl Acad Sci U S A 106:21848–21853. doi:10.1073/pnas.0911354106 20007369 PMC2799887

[68] de Souza FG, Abrahão JS, Rodrigues RAL. 2021. Comparative analysis of transcriptional regulation patterns: understanding the gene expression profile in Nucleocytoviricota. Pathogens 10:935. doi:10.3390/pathogens10080935 34451399 PMC8400408

[69] Ha AD, Moniruzzaman M, Aylward FO. 2021. High transcriptional activity and diverse functional repertoires of hundreds of giant viruses in a coastal marine system. mSystems 6:e0029321. doi:10.1128/mSystems.00293-21 34254826 PMC8407384

[70] Farzad R, Ha AD, Aylward FO. 2022. Diversity and genomics of giant viruses in the North Pacific subtropical gyre. Front Microbiol 13:1021923. doi:10.3389/fmicb.2022.1021923 36504832 PMC9732441

[71] Talbert PB, Armache KJ, Henikoff S. 2022. Viral histones: pickpocket’s prize or primordial progenitor?. Epigenetics Chromatin 15:21. doi:10.1186/s13072-022-00454-7 35624484 PMC9145170

[72] Koonin EV, Yutin N. 2010. Origin and evolution of eukaryotic large nucleo-cytoplasmic DNA viruses. Intervirology 53:284–292. doi:10.1159/000312913 20551680 PMC2895762

[73] Moureau G, Cook S, Lemey P, Nougairede A, Forrester NL, Khasnatinov M, Charrel RN, Firth AE, Gould EA, de Lamballerie X. 2015. New insights into flavivirus evolution, taxonomy and biogeographic history, extended by analysis of canonical and alternative coding sequences. PLoS One 10:e0117849. doi:10.1371/journal.pone.0117849 25719412 PMC4342338

[74] Parry R, Asgari S. 2019. Discovery of novel crustacean and cephalopod flaviviruses: insights into the evolution and circulation of flaviviruses between marine invertebrate and vertebrate hosts. J Virol 93:e00432-19. doi:10.1128/JVI.00432-19 31068424 PMC6600200

[75] Wang S, Liu H, Zu X, Liu Y, Chen L, Zhu X, Zhang L, Zhou Z, Xiao G, Wang W. 2016. The ubiquitin-proteasome system is essential for the productive entry of Japanese encephalitis virus. Virology 498:116–127. doi:10.1016/j.virol.2016.08.013 27567260

[76] Mayer D, Thayer TM, Hofmann MA, Tratschin JD. 2003. Establishment and characterisation of two cDNA-derived strains of classical swine fever virus, one highly virulent and one avirulent. Virus Res. 98:105–116. doi:10.1016/j.virusres.2003.08.020 14659557

[77] Simmonds P, Becher P, Bukh J, Gould EA, Meyers G, Monath T, Muerhoff S, Pletnev A, Rico-Hesse R, Smith DB, Stapleton JT, Ictv Report Consortium. 2017. ICTV virus taxonomy profile: flaviviridae. J Gen Virol 98:2–3. doi:10.1099/jgv.0.000672 28218572 PMC5370391

[78] Ter Horst AM, Nigg JC, Dekker FM, Falk BW. 2019. Endogenous viral elements are widespread in arthropod genomes and commonly give rise to PIWI-interacting RNAs. J Virol 93:e02124-18. doi:10.1128/JVI.02124-18 30567990 PMC6401445

[79] Veglia AJ, Bistolas KSI, Voolstra CR, Hume BCC, Ruscheweyh H-J, Planes S, Allemand D, Boissin E, Wincker P, Poulain J, et al.. 2023. Endogenous viral elements reveal associations between a non-retroviral RNA virus and symbiotic dinoflagellate genomes. Commun Biol 6:566. doi:10.1038/s42003-023-04917-9 37264063 PMC10235124

[80] Gilbert C, Belliardo C. 2022. The diversity of endogenous viral elements in insects. Curr Opin Insect Sci 49:48–55. doi:10.1016/j.cois.2021.11.007 34839030

[81] Terzian C, Pélisson A, Bucheton A. 2001. Evolution and phylogeny of insect endogenous retroviruses. BMC Evol Biol 1:3. doi:10.1186/1471-2148-1-3 11591216 PMC57737

[82] Wu N, Zhang P, Liu W, Wang X. 2018. Sogatella furcifera hepe-like virus: first member of a novel Hepeviridae clade identified in an insect. Virus Res. 250:81–86. doi:10.1016/j.virusres.2018.03.018 29605729

[83] Jacquat AG, Ulla SB, Debat HJ, Muñoz-Adalia EJ, Theumer MG, Pedrajas MDG, Dambolena JS. 2022. An in silico analysis revealed a novel evolutionary lineage of putative mitoviruses. Environ Microbiol 24:6463–6475. doi:10.1111/1462-2920.16202 36085554

[84] Cross ST, Maertens BL, Dunham TJ, Rodgers CP, Brehm AL, Miller MR, Williams AM, Foy BD, Stenglein MD. 2020. Partitiviruses infecting Drosophila melanogaster and Aedes aegypti exhibit efficient biparental vertical transmission. J Virol 94:e01070-20. doi:10.1128/JVI.01070-20 32759315 PMC7527066

[85] Canuti M, Large G, Verhoeven JTP, Dufour SC. 2022. A novel Iridovirus discovered in deep-sea carnivorous sponges. Viruses 14:1595. doi:10.3390/v14081595 35893660 PMC9330688

[86] Feng Y, Krueger EN, Liu S, Dorman K, Bonning BC, Miller WA. 2017. Discovery of known and novel viral genomes in soybean aphid by deep sequencing. Phytobiomes J 1:36–45. doi:10.1094/PBIOMES-11-16-0013-R

[87] Gáspár G, De Clercq E, Neyts J. 2002. Human herpesvirus 8 gene encodes a functional thymidylate synthase. J Virol 76:10530–10532. doi:10.1128/jvi.76.20.10530-10532.2002 12239332 PMC136588

[88] Wnuk SF, Robins MJ. 2006. Ribonucleotide reductase inhibitors as anti-herpes agents. Antiviral Res. 71:122–126. doi:10.1016/j.antiviral.2006.03.002 16621038

[89] Dufour F, Bertrand L, Pearson A, Grandvaux N, Langelier Y. 2011. The ribonucleotide reductase R1 subunits of herpes simplex virus 1 and 2 protect cells against poly(I · C)-induced apoptosis. J Virol 85:8689–8701. doi:10.1128/JVI.00362-11 21697465 PMC3165841

[90] Rosani U, Gaia M, Delmont TO, Krupovic M. 2023. Tracing the invertebrate herpesviruses in the global sequence datasets. Front Mar Sci 10:1159754. doi:10.3389/fmars.2023.1159754

[91] Holt CC, Boscaro V, Van Steenkiste NWL, Herranz M, Mathur V, Irwin NAT, Buckholtz G, Leander BS, Keeling PJ. 2022. Microscopic marine invertebrates are reservoirs for cryptic and diverse protists and fungi. Microbiome 10:161. doi:10.1186/s40168-022-01363-3 36180959 PMC9523941

[92] Mouritsen KN, Poulin R. 2002. Parasitism, community structure and biodiversity in intertidal ecosystems. Parasitology 124 Suppl:S101–17. doi:10.1017/s0031182002001476 12396219

[93] Li Y, Shen X-X, Evans B, Dunn CW, Rokas A. 2021. Rooting the animal tree of life. Mol Biol Evol. 38:4322–4333. doi:10.1093/molbev/msab170 34097041 PMC8476155

[94] Weitz JS, Poisot T, Meyer JR, Flores CO, Valverde S, Sullivan MB, Hochberg ME. 2013. Phage-bacteria infection networks. Trend Microb 21:82–91. doi:10.1016/j.tim.2012.11.003 23245704

[95] Valverde S, Vidiella B, Montañez R, Fraile A, Sacristán S, García-Arenal F. 2020. Coexistence of nestedness and modularity in host-pathogen infection networks. Nat Ecol Evol 4:568–577. doi:10.1038/s41559-020-1130-9 32152533

[96] Tomlinson KR, Pablo-Rodriguez JL, Bunawan H, Nanyiti S, Green P, Miller J, Alicai T, Seal SE, Bailey AM, Foster GD. 2019. Cassava brown streak virus Ham1 protein hydrolyses mutagenic nucleotides and is a necrosis determinant. Mol Plant Pathol 20:1080–1092. doi:10.1111/mpp.12813 31154674 PMC6640186

[97] Rey-Campos M, González-Vázquez LD, Novoa B, Figueras A. 2023. Metatranscriptomics unmasks mollusca virome with a remarkable presence of rhabdovirus in cephalopods. Front Mar Sci 10:1209103. doi:10.3389/fmars.2023.1209103

[98] Dunn CW, Hejnol A, Matus DQ, Pang K, Browne WE, Smith SA, Seaver E, Rouse GW, Obst M, Edgecombe GD, Sørensen MV, Haddock SHD, Schmidt-Rhaesa A, Okusu A, Kristensen RM, Wheeler WC, Martindale MQ, Giribet G. 2008. Broad phylogenomic sampling improves resolution of the animal tree of life. Nature 452:745–749. doi:10.1038/nature06614 18322464

[99] Kocot KM, Struck TH, Merkel J, Waits DS, Todt C, Brannock PM, Weese DA, Cannon JT, Moroz LL, Lieb B, Halanych KM. 2017. Phylogenomics of Lophotrochozoa with consideration of systematic error. Syst Biol 66:256–282. doi:10.1093/sysbio/syw079 27664188

[100] Higgins RP. 1978. Echinoderes gerardi n. sp. and E. riedli (Kinorhyncha) from the Gulf of Tunis. Trans Amer Micr Soc 97:171. doi:10.2307/3225589

[101] Neuhaus B, Higgins RP. 2002. Ultrastructure, biology, and phylogenetic relationships of Kinorhyncha. Integr Comp Biol 42:619–632. doi:10.1093/icb/42.3.619 21708758

[102] Dolja VV, Koonin EV. 2018. Metagenomics reshapes the concepts of RNA virus evolution by revealing extensive horizontal virus transfer. Virus Res. 244:36–52. doi:10.1016/j.virusres.2017.10.020 29103997 PMC5801114

